# The actinin family proteins: biological function and clinical implications

**DOI:** 10.1186/s13578-015-0039-5

**Published:** 2015-08-25

**Authors:** Hung-Ying Kao

**Affiliations:** Department of Biochemistry, Case Western Reserve University, 10900 Euclid Avenue, Cleveland, OH 44106-4935 USA

The alpha actinin (ACTN) family proteins are actin-bundling proteins that are evolutionarily conserved and possess several functional domains. Vertebrates including humans express four unique actinin genes that display distinct expression patterns and biochemical properties. Mutations in each of these actinins are associated with tissues-specific abnormalities. ACTN1 and ACTN4 are ubiquitously expressed and their major spliced isoforms harbor calcium-sensitive EF hands, while the expression of ACTN2 and ACTN3 are primarily restricted to muscle and are calcium-insensitive.

Due to their ability to bind filamentous actin, ACTNs have been shown to regulate cytokinesis, cell adhesion, spreading, migration and signaling. In addition, a fraction of ACTN4 is localized in the nucleus where it interacts with transcription factors, histone modifying enzymes and chromatin remodeling proteins in order to stimulate transcription [1–3]. Notably, all ACTNs harbor an evolutionarily conserved motif,—LXXLL—(L, leucine, X, any amino acids), which is required for interaction with the nuclear receptor family of transcription factors [3]. Indeed, ACTN1 and ACTN2 have been shown to enhance nuclear receptor-mediated transcription [1, 4]. Thus, ACTNs join the list of several other cytoskeletal proteins that are capable of shuttling between the cytoplasm and the nucleus to control cell function [5].

In this thematic review series, we feature recent progress in our understanding of the biological and the pathological roles of the ACTNs and genetic lesions in this protein family that are associated with human diseases.

Murphy and Young [6] first summarize genetic studies of actinin in invertebrates and cast an evolutionary perspective of this unique actin-binding protein family. They detail the similarities and differences among the 4 vertebrate ACTNs and provide an insightful discussion of actinin-related genetic disorders with primary focus on ACTN1, 2 and 3. For example, mutations in *ACTN1* are associated with dominantly-inherited congenital macrothrombocytopenia (CMPT), a rare blood disorder, while mutations in *ACTN2* are linked to hypertrophic cardiomyopathy (HCM). Interestingly, disease-associated *ACTN1* and *ACTN2* mutations are only found in the actin-binding domain, emphasizing the physiological significance of this functional domain. Surprisingly, *ACTN3* is not essential and individuals with *ACTN3* null alleles, due to a homozygous premature stop codon, are found in 16 % of world population. Interestingly, wild-type allele is found to be associated with better sprint and power performance in Caucasians.

ACTN4 was initially identified in a screen for antigens to which antibodies strongly reacted in highly invasive breast cancer [7]. Honda reviews recent literature on the link between *ACTN4* amplification, overexpression and spliced variants that predict metastatic potency in several types of tumors including breast, prostate, colon and lung [8]. He also summarizes evidence supporting a role of ACTN4 in transcriptional regulation by nuclear receptors and NF-κB and its link to tumorigenesis. Lastly, Honda suggests that ACTN4 may be a useful biomarker for evaluating treatment option in metastatic breast cancer.

An elegant human genetics study has linked *ACTN4* mutations to the kidney disorder focal segmental glomerular sclerosis (FSGS) [9]. This finding pioneered a novel concept that mutations in genes encoding cytoskeletal proteins in podocytes may be linked to glomerulopathy. Indeed, mutations of several cytoskeletal proteins have been subsequently found in patients with familial FSGS [10]. Interestingly, reduced expression of ACTN4 protein in kidney was also found in sporadic FSGS, minimal change disease and IgA nephropathy. Feng et al., thoroughly discusses the clinical presentation and biochemical and biophysical properties of disease causing *ACTN4* mutations as well as related animal studies [11]. They further provide potential mechanisms underlying *ACTN4*-linked glomerulopathy.

The physiological function of ACTN4 is unlikely limited to bundling actin and co-activating transcription. It is likely to also be a signaling molecule that transmits extracellular signalings to influence cell growth and differentiation. The intent of this thematic series is to summarize recent discoveries on actinin function and ACTN-related diseases and highlight future challenges in ACTN research.
